# Kinetics of Proton Transport into Influenza Virions by the Viral M2 Channel

**DOI:** 10.1371/journal.pone.0031566

**Published:** 2012-03-06

**Authors:** Tijana Ivanovic, Rutger Rozendaal, Daniel L. Floyd, Milos Popovic, Antoine M. van Oijen, Stephen C. Harrison

**Affiliations:** 1 Department of Biological Chemistry and Molecular Pharmacology, Harvard Medical School, Boston, Massachusetts, United States of America; 2 Department of Molecular, Cellular and Developmental Biology, University of Colorado, Boulder, Colorado, United States of America; 3 Department of Electrical Engineering, University of Colorado, Boulder, Colorado, United States of America; 4 Howard Hughes Medical Institute, Harvard Medical School, Boston, Massachusetts, United States of America; University of Edinburgh, United Kingdom

## Abstract

M2 protein of influenza A viruses is a tetrameric transmembrane proton channel, which has essential functions both early and late in the virus infectious cycle. Previous studies of proton transport by M2 have been limited to measurements outside the context of the virus particle. We have developed an *in vitro* fluorescence-based assay to monitor internal acidification of individual virions triggered to undergo membrane fusion. We show that rimantadine, an inhibitor of M2 proton conductance, blocks the acidification-dependent dissipation of fluorescence from a pH-sensitive virus-content probe. Fusion-pore formation usually follows internal acidification but does not require it. The rate of internal virion acidification increases with external proton concentration and saturates with a pK_m_ of ∼4.7. The rate of proton transport through a single, fully protonated M2 channel is approximately 100 to 400 protons per second. The saturating proton-concentration dependence and the low rate of internal virion acidification derived from authentic virions support a transporter model for the mechanism of proton transfer.

## Introduction

Influenza virus depends on pH gradients and their regulation at several steps during entry and assembly. The virus enters cells by fusion of the viral lipid envelope with the target-cell endosomal membrane. The low endosomal pH triggers a series of conformational rearrangements in the viral hemagglutinin (HA), which facilitate apposition and merger of the two membranes, in a sequence of events that include hemifusion (mixing of outer leaflets) and subsequent opening of a small membrane pore, which expands to release the viral genome into the target-cell cytosol. Release of the genome also requires internal acidification of the virion, to dissociate the viral matrix protein, M1, from the viral RNPs [1]. The viral M2 protein, a tetrameric proton channel [2], [3], conducts protons from the endosomal lumen into the virion. In the trans-Golgi network of infected cells, M2 also prevents low-pH inactivation of HA following activation of the precursor, HA0, by furin cleavage to HA1 and HA2 [4], and it appears to have yet a further role during membrane scission, the final step of virus budding [5]. A small effect of M2 activity on the kinetics of hemifusion has led to the proposal that internal virion acidification can accelerate membrane remodeling driven by HA conformational rearrangements in the context of an intact virion [6], [7]. Those observations suggest that the effect of internal acidification on the kinetics of fusion-pore formation might be more substantial, but this possibility has not been explored.

The M2 polypeptide chain of 97 residues passes once through the membrane. At the heart of the channel is a histidine tetrad, implicated in both proton selectivity and low-pH channel activation [8]. Solid-state NMR measurements have shown that the first two histidines of the tetrad are protonated with pK_a_∼8.2; the remaining two pK_a_s are 6.3 and <5 [9]. These results have led to the suggestion that cooperative protonation of two of the four histidines results in symmetric, strongly hydrogen-bonded, imidazole pairs and that addition of charge to the third and fourth imidazole side chains activates the channel; they leave open the question, whether histidine protonation is a step in proton transfer from one side of the membrane to the other. Molecular dynamics simulations have led to opposing models, describing M2 either as a simple proton channel or as a proton transporter [10], [11], [12]. Measurements of the kinetics of proton transfer through M2 channels reconstituted in liposomes have offered support to the transporter model, by revealing Michaelis-Menten-like dependence of conductivity on pH, with two saturation steps (pK_app_ 6.25 and 4.7) and slow transport rates (14 protons/channel/sec between pH 7.2 and 5.2; 40 protons/channel/sec between pH 5 and 3.4) [13]. This model has found additional recent support in solid-state-NMR-based functional-dynamics measurements of M2-channel histidines in synthetic membranes [14].

To study M2 activity in the context of an intact virion, to determine whether M2 activity influences fusion, and to determine the relative rates of internal acidification and fusion-pore formation, we have adapted our single-virion fusion assay to include measurements of the kinetics of internal acidification. We find that internal acidification has no influence on fusion kinetics, but that a drop in internal pH nonetheless generally precedes fusion-pore formation. The results support a transporter model, in which the channel histidines participate in moving protons down a concentration gradient.

## Results

### Dissipation of fluorescein signal by virion acidification

We followed fusion of influenza virus particles with target membranes as previously described [15]. To monitor internal acidification, we labeled the virion interior with fluorescein, a pH-sensitive fluorophore with pKa = 6.4.

**Figure 1:**
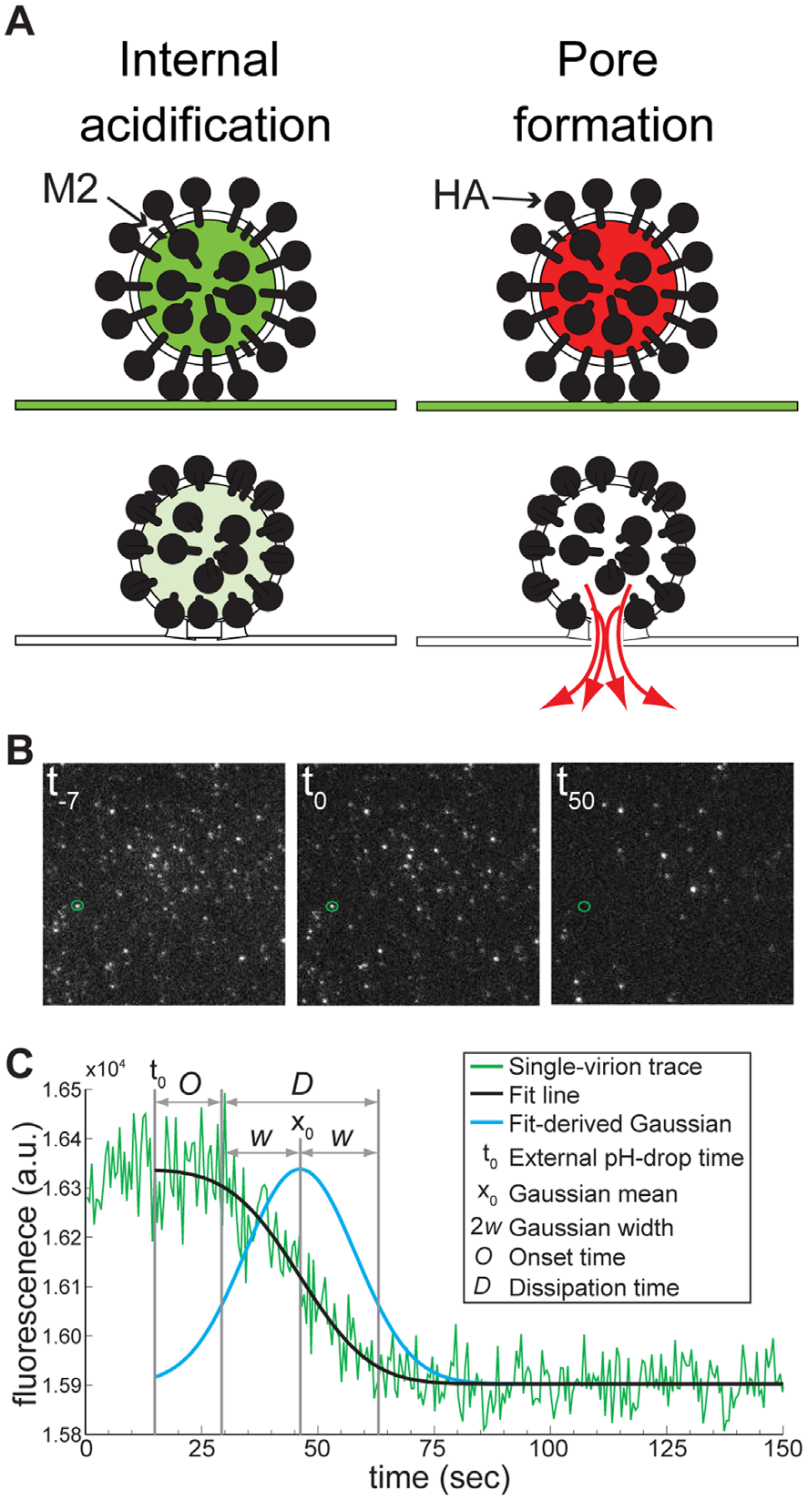
Fluorescein-based single-virion assay for the study of internal virion acidification. A) Loading of virions with pH-sensitive (fluorescein, green) or pH-stable (SRB, red) fluorophores for detection of M2-mediated internal virion acidification (left) or HA-mediated viral membrane fusion (right; 15). Target synthetic bilayers incorporate sialic-acid receptors and a fluorescein pH indicator. B) Selected frames from a representative time-lapse movie monitoring fluorescein-loaded virions at pH 4.5 (t_−7_, 7 sec before the pH drop; t_0_, the time of the pH drop; t_50_, 50 seconds after the pH drop). A subsection (∼1/6) of the imaged ∼70×140 µm area is shown. The green circle marks a single virion analyzed further in C. C) A representative fluorescence intensity-versus-time single-virion trace (green line), the model used to fit the data (inset), best-fit line (black) and fit-derived Gaussian (blue). Fit-derived parameters: t_c_, Gaussian mean or the time at which the transition is half complete; *w*, Gaussian width, or 2^1/2^ x the standard deviation of the underlying distribution. Onset time: *O* = *t_o_*−*(t_c_−w)*. Dissipation time: *D = 2w*, centered at *t_c_*.

We mounted target membranes incorporating influenza virus receptor and external fluorescein pH probe on dextran-functionalized glass cover slips over a TIRF objective [15]. We attached dye-loaded virus particles to these membranes and reduced the pH of the exterior to 4.5 immediately after the onset of imaging. Figure 1B shows selected frames from a time-lapse movie monitoring fluorescein-loaded virions. Before the external pH drop we see both the labeled virions (bright spots) and the external pH probe (diffuse background fluorescence) (Figure 1B, left). At the point we define as the reaction start time (*t_o_*), more than 90% of the external-pH-probe fluorescence has dissipated (Figure 1B, middle), and dissipation of fluorescence from virions follows (Figure 1B, right).

**Figure 2 pone-0031566-g002:**
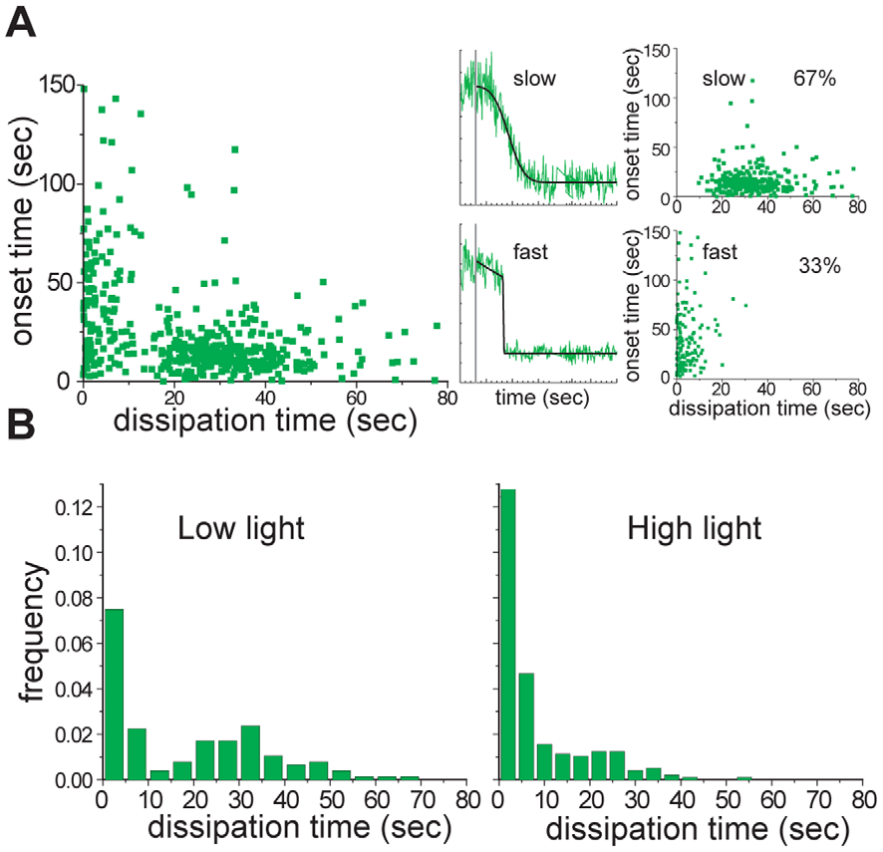
Light-induced fast dissipation of fluorescein. A) Scatter plot of dissipation versus onset times for individual virions at pH 4.5 and under low-power illumination (all dissipating particles, left). We clustered the observations by inspection into fast- and slow-dissipating populations, by looking at each particle trace and categorizing based on the apparent sharpness of the transition (sorted subpopulations, right). Representative virion traces from slow- and fast-dissipating subpopulations are shown in the middle (raw data, green; best-fit line, black). B) Dissipation-time frequency histograms under low- and high-power illumination.

A scatter plot, in which each virus particle is represented by a point positioned according to its dissipation and onset times, reveals two distinct clusters (Figure 2A). Experiments described in the caption to Fig. 2 show that the rapidly dissipating subpopulation, which is rimantidine resistant, comes from photodamage. We therefore performed all measurements under low-light conditions and separately analyzed slow- and fast-dissipating subpopulations.

### Distinct kinetics of fluorescein and SRB signal dissipation

**Figure 3 pone-0031566-g003:**
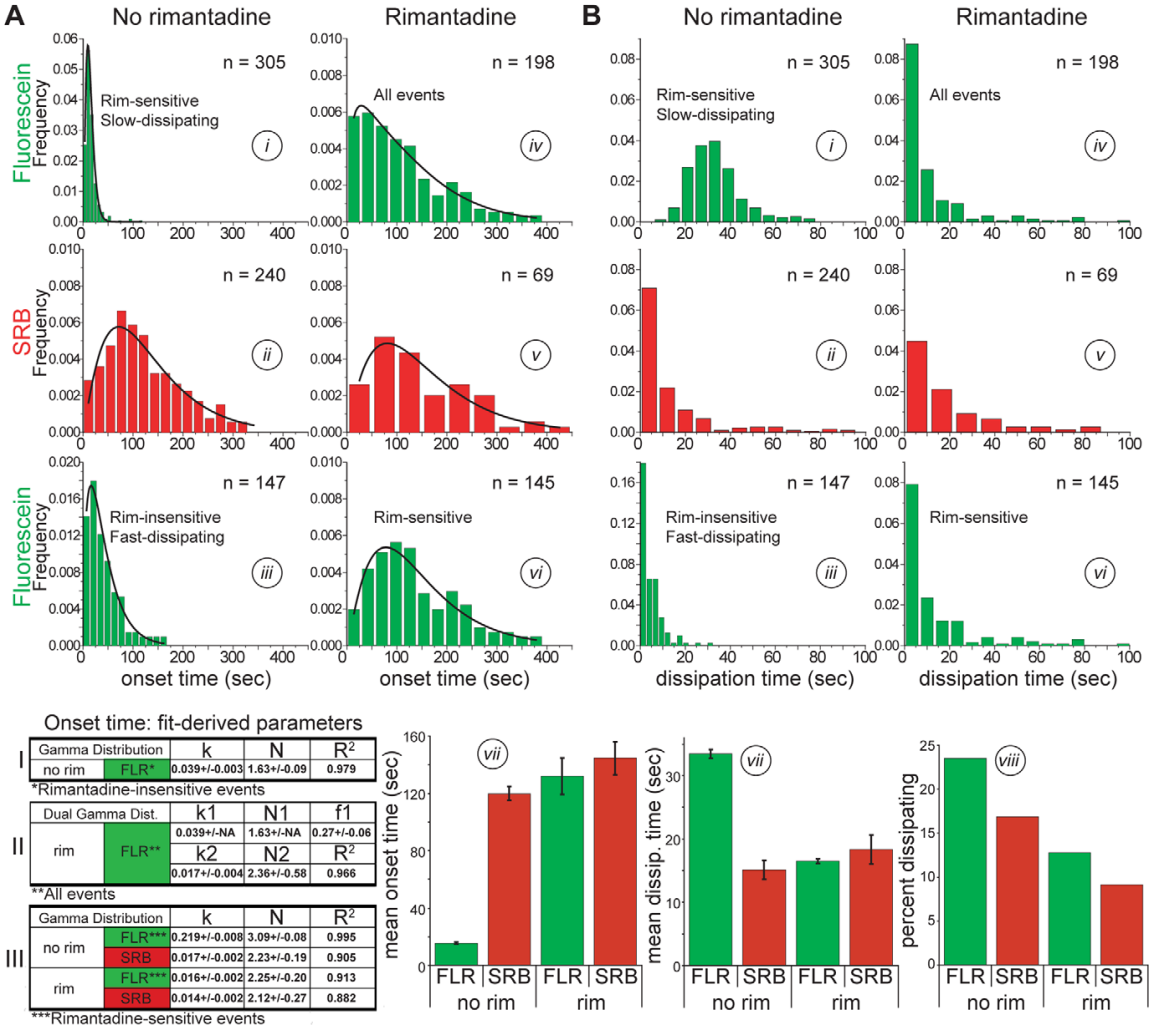
Comparison of fluorescein and SRB signal-dissipation kinetics. A) Onset times, and B) dissipation times (plots i to vii) of fluorescein (green) and SRB (red) signals at pH 4.5, in the absence of rimantadine (plots i to iii) or in its presence (plots iv to vi). Plots i and iii, from experiments carried out in the absence of rimantidine, show fluorescein dissipation kinetics for the “rimantidine-sensitive” and “rimantidine-insensitive” subpopulations, respectively; these subpopulations were identified and sorted as described in the caption to Fig. 2. The kinetics of the latter subpopulation (plot iii, Table I) were used to estimate the contribution of this component to the overall kinetics in the presence of rimantidine (plot iv, Table II, parameter f1). Subtraction of this contribution yielded the kinetics of rimantidine-sensitive events in the presence of the drug (plot vi, Table III). See Methods for details. Onset times in A were fit with either a single gamma distribution (plots i, ii, iii, v and vi and tables I and III) or a dual gamma distribution (plot iv and table II) (see Methods). Plot vii shows mean times calculated from data shown in plots i, ii, vi and v, respectively. Error bars represent the standard error of the mean, or, in the case of fluorescein-signal dissipation in the presence of rimantadine, errors estimated from the error of the fit-derived f1 parameter (see Methods). Plot viii in B shows the percent of the overall virion population represented respectively by plots i, ii, vi, and v.

Protonation of fluorescein and fusion-pore opening will both lead to a drop in recorded fluorescence over time. We compared the kinetics of fluorescence dissipation from virions singly loaded with either dye. The kinetics of the two processes were unchanged, whether assayed in the same or in separate experiments, and we therefore combined data from multiple experiments (Figure 3). The mean onset time for loss of the fluorescein signal (15.3 sec) was much shorter than for loss of SRB (119.9 sec) (Figure 3A, plot vii, and compare plots i and ii); the mean dissipation time for fluorescein (33.5) was longer than for SRB (15.2 sec) (Figure 3B, plot vii, and compare plots i and ii), consistent with our assumption in what follows that they represent separate processes.

### Rimantadine delays internal fluorescein-signal dissipation

We pretreated the fluorescein- and the SRB-loaded virions with 10 µM rimantadine and assayed the kinetics of fluorescent-signal dissipation in the continuous presence of rimantadine. Rimantadine largely prevented the early dissipation of fluorescein signal (Figure 3A, compare plots i and iv). The onset-time distributions for fluorescein and SRB in the presence of rimantadine were not identical, as the fluorescein data showed relative enrichment in the bins corresponding to shorter onset times (Figure 3A, compare plots iv and v). We interpreted this enrichment as a contribution from the light-induced, rimantadine-insensitive subpopulation, which we could estimate and subtract from the overall distribution (see Fig. 3A and Methods). Resulting data show that fluorescein dissipation in the presence of rimantadine is indistinguishable from SRB dissipation under the same conditions (Figure 3, table III, plot vii, and compare plots v and vi). A parallel analysis of dissipation-time distributions yielded identical conclusions. Rimantadine treatment converted the fluorescein-dissipation kinetics to that of SRB (Figure 3B, plot vii, and compare plots i, v and vi). Thus, loading of influenza virions with fluorescein enables unambiguous detection of low-pH-triggered, M2-mediated internal virion acidification.

### Virion acidification is not required for fusion

Rimantadine treatment had no significant effect on either the onset or the dissipation times of the SRB signal (Figures 3A and 3B, plot vii and compare plots ii and v, and Figure 3A, table III). Internal virion acidification therefore has no effect on the kinetics of small fusion pore opening and hence on the HA conformational transition. We did observe a modest (1.6-fold) decrease in the fraction of virions (average for fluorescein- and SRB-loaded virions) that lost fluorescence in the presence of rimantadine relative to those (SRB-loaded virions) that fused in its absence (Figure 3B, plot viii). We believe that this loss of apparent fusion competence might come from an effect of rimantadine treatment on properties of the virion other than those required for the low-pH induced conformational change in HA, such as perturbations in the viral membrane that may or may not result from a specific effect on M2.

### pH dependence of internal virion acidification

**Figure 4:**
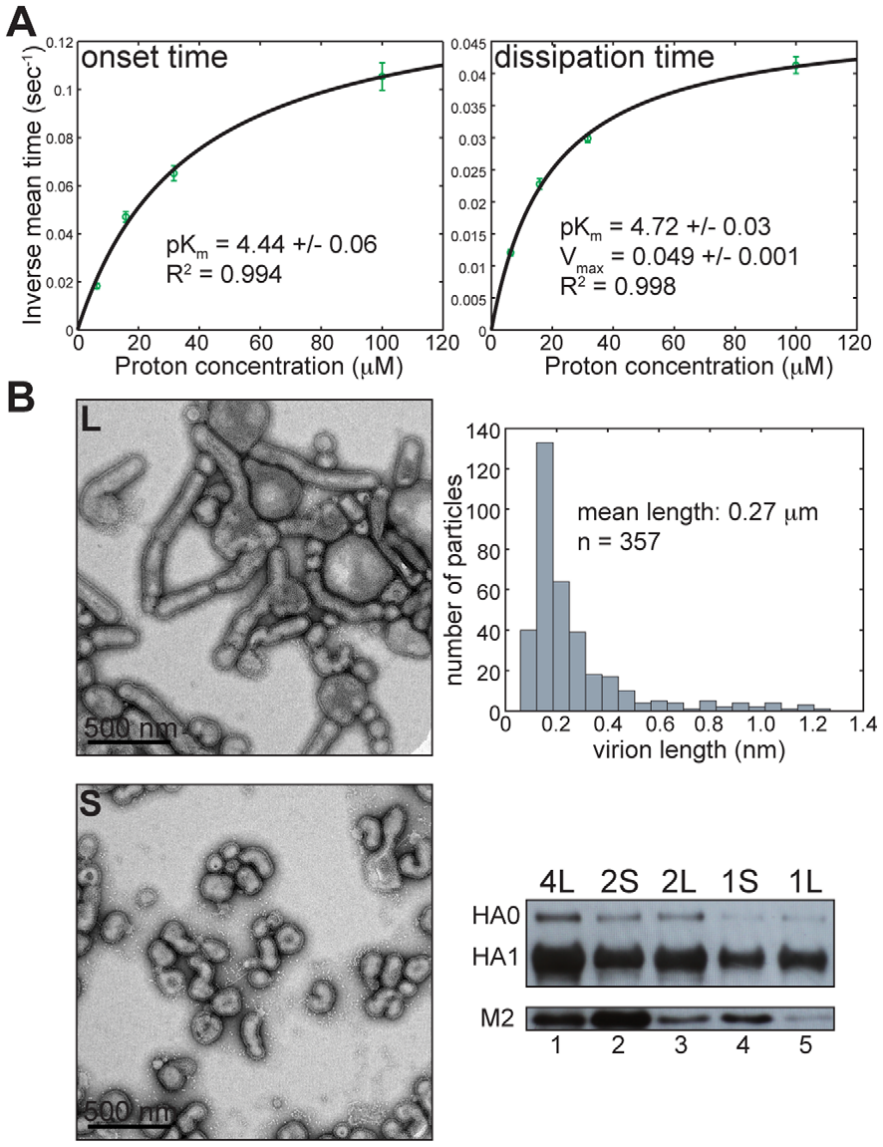
Kinetics of internal virion acidification. A) Proton-concentration dependence of inverse onset (left) and dissipation times (right). Error bars are propagated from the standard error of the mean (SEM) of measured onset and dissipation times as SEM(mean time)/(mean time)^2^. B) Number of M2 channels per virion does not scale with virion size. *Upper and lower left* – representative negative-stain electron micrographs of long (L) and short (S) virion fractions. All kinetic measurements in Figures 1 to [Fig pone-0031566-g002] [Fig pone-0031566-g003] 4 used the long-virion fraction. *Upper right* – long-virion length histogram distribution as estimated from multiple negative stain electron micrographs (see Materials and Methods). *Lower right* – SDS-PAGE of two-fold serial dilutions of long (4L, 2L, 1L) and short (2S, 1S) virion fractions and immunoblotting using anti-HA1 antibody (upper panel, HA0 and HA1) or anti-M2 antibody (lower panel, M2).

To probe the mechanism of proton conductance by M2 we determined the onset time and duration of fluorescein signal dissipation over a range of proton concentrations from 6 to 100 µM (pH range 5.2 to 4). As expected for pH-activated processes, both onset and dissipation times decrease with increased proton concentration. Overall rates for both onset and dissipation (inverse mean onset and dissipation times) obey Michaelis-Menten-like kinetics; fitting the rate data to
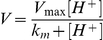
yields pK_m_ values of 4.44+/−0.06 (R^2^ = 0.994) and 4.72+/−0.03 (R^2^ = 0.998), respectively (Figure 4A). These values correspond to the measured pK_a_ of the fourth protonation event in the channel histidine tetrad [9], and the observed pH dependence closely mimics that measured for the purified M2 protein reconstituted in liposomes and triggered to conduct protons at pHs ranging from 5 to 3.4 [13]. By distinguishing between onset times (related to the probability of channel activation) and dissipation times (related to both the probability of channel activation and proton flux through an activated channel) and by showing essentially the same dependence of rates on proton concentration, we conclude that channel activation and proton conductance are related events, limited by the concentration of the fully protonated channel histidines.

### Slow rate of proton transport into virions by M2

To calculate an absolute rate of proton passage through viral-membrane associated M2 we estimated the number of M2 channels per virion as well as the number of internal viral-protein- and fluorescein-associated titratable groups for the virion preparation used in our kinetic measurements (the long fraction of MDCK-cell grown virus) (see Materials and Methods for detailed description). So that our estimates of the number of internal titratable groups would represent an upper limit, we assumed that all histidine residues within viral proteins M1 and NP have pK_a_s spanned by the starting and the target pH in our experiments.

An early estimate of M2 content gave 14 to 68 M2 molecules, or 4 to 17 M2 channels in a spherical virion of diameter ∼100 nm [16]. Recent work implicating M2 in the final steps of influenza virus budding has suggested that the number of M2 channels per virion is independent of virion size, but detection of M2 in these experiments by antibody binding was limited to intact virions, leaving open a possibility that additional M2 epitopes were masked by dense packing of HA on the virion surface [5]. We compared by SDS-PAGE and immunoblotting the long and short virion fractions for relative levels of HA1 and M2 per virion. When comparable levels of HA1 from short and long virions were loaded, there was much less M2 in the long-virion lane (Figure 4B, lower right, compare lanes 2 and 3 and lanes 4 and 5). Even when we loaded substantially more long virion-associated HA1 than short-virion associated HA1, we could not equalize the levels of long and short virion-associated M2. Thus, the number of M2 channels per virion does not scale with virion size (Figure 4B, lower right, compare lanes 1 and 2 and lanes 3 and 4), consistent with the observation that a short-virion preparation of egg-derived A/Udorn/72 showed more rapid internal acidification than did our MDCK-cell grown long-virion fraction expressing genetically identical M2 (Figure S1).

By multiplying the *V*_max_ obtained from the Michaelis-Menten fit of the fluorescein dissipation rate (0.049 sec^−1^+/−0.001) (Figure 4A, right) by our estimate for the number of internal titratable groups (31,000) (see Materials and Methods) and dividing by the published estimate of M2 channels per virion (4 to 17) [16], we obtain a range of rates of proton transport through M2 of ∼100 to ∼400 protons/channel/second. This is consistent with an estimate for the rate of proton conductance by purified M2 reconstituted in liposomes in the same pH regime [13].

## Discussion

**Figure 5:**
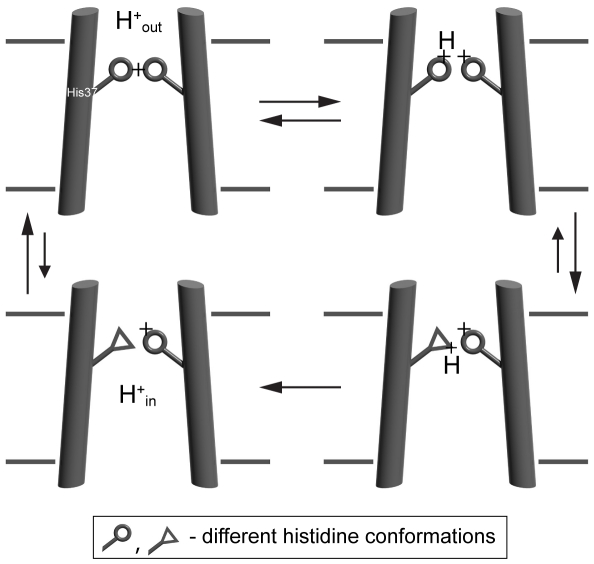
A transporter model for M2-mediated proton transport into virions. Conformational changes in the channel histidines allow their protonation on the virion exterior and deprotonation on the virion interior facilitating transport of protons down the concentration gradient.

Our single-virion assay permits measurement of M2 channel activity in the context of authentic influenza virions triggered to undergo membrane fusion. The experiments are consistent with two predictions of a transporter model (Figure 5) for passage of protons into the virion interior through M2 channels: 1) Michealis-Menten-like dependence of onset and dissipation rates with pK_m_s coinciding with the pK_a_ of the fourth channel histidine and 2) slow rate of proton transport. Our kinetic measurements of M2 channel activity on authentic virions complement recent findings from purified M2 protein reconstituted in liposomes and triggered to conduct protons in the pH range from 5 to 3.4 [13]. That work identified a second pseudosaturation step (pK_m_ 6.25) corresponding to the measured pK_a_ of the third protonation event in the channel histidine tetrad. Fluorescein is not a useful probe for detecting internal virion pH changes in this second regime, because of a combination of factors that include the very slow rate of proton transport in this regime, the photobleaching of fluorescein after prolonged laser-light exposure, and the almost identical pK_a_s of fluorescein (∼6.4) and the third channel histidine (∼6.3). Nonetheless, the similar rates of proton transport and the similar dependence of these rates on proton concentration are strong evidence that the activity of M2 in authentic virions is insensitive to the presence of other viral proteins or the membrane remodeling induced by HA conformational changes during fusion.

Parallel measurements of kinetics of internal virion acidification and membrane pore-formation allow direct comparisons of the relative timing of the two processes. The onset of internal virion acidification generally precedes that of pore formation, but the latter does not require acidification to proceed at its normal rate. The kinetics of pore formation were unchanged in the presence of 10 µM rimantadine, which blocked internal virion acidification. Thus, there appears to be no direct molecular signal linking acidification to pore formation. Nonetheless, because fusion with the endosomal membrane will equilibrate the virion pH with that of the cytosol, release of nucleocapsid from M1 requires that proton transport be rapid enough to reduce the internal pH before a fusion pore opens. Our results show that this relationship holds. We do observe, however, a small overlap in the distribution of pore-formation onset times with that of acidification onset times. During cell entry, a corresponding fraction of early pore-formation events might include non-productive infections, if pore formation were to occur before an adequate drop in internal pH. Because of the relatively small size of our probe (SRB MW is 559), our observations leave open the possibility that internal virion acidification could influence the transition from initial opening of a small fusion pore to widening of that pore into a channel large enough for genome translocation. In addition to showing the mutual independence of membrane fusion and internal virion acidification, the methods described here provide a format for studying specific inhibition of either of these low-pH-induced processes, both of which are essential for productive viral entry.

## Materials and Methods

### Virions

#### Preparation

Plasmids for generation of influenza A/Udorn/72 were kindly provided by Robert Lamb. To generate a construct that replaces Udorn HA with the HA of X31 influenza, we swapped the Udorn HA open reading frame (ORF) in the pHH21 plasmid background [17] with that of X31 by standard cloning techniques. To generate X31-HA cDNA, we extracted RNA from egg-grown X31 virions, reverse transcribed and amplified it in a single reaction (One-step RT-PRC, Qiagen). We used primers specific for the terminal sequences of the X31 HA ORF, each with both a flanking linker and the *Bsm* B1 restriction site, digestion of which generates the product that terminates precisely with the ORF's ends. Introduction of the corresponding *Bsm* B1 sites in the pHH21-UdornHA construct allowed swapping of the two ORFs after a *Bsm* B1 digest (NEB) and a single ligation reaction (T4 DNA ligase, NEB). The protocol for recombinant influenza rescue was based on that developed by Neumann et al. [18] and later adapted for A/Udorn/72 by Takeda et al. [17]. We generated virus expressing X31 HA in an otherwise Udorn-derived background (Udorn-X31HA), then plaque purified and amplified it by two passages on MDCK cells (ATCC strain CCL-34 kindly provided by Adolfo Garcia-Sastre) at low multiplicity of infection (0.001 PFU/cell) in the presence of 1 µg/ml TPCK-trypsin (Sigma). In the final step we infected MDCK cells with 3 PFU/cell and harvested the virus-containing supernatant before significant cytopathic effect took place (between 13 and 16 h post infection) to maximize the purity of our virus preparation. We clarified the supernatant by centrifugation at 2,800× g for 15 minutes, then pelleted the virus by centrifugation at 100,000× g for 1.5 h. We resuspended viral pellets in HNE20 (20 mM HepesNaOH pH 7.4, 150 mM NaCl, 1 mM EDTA) and purified them by sequential centrifugation, first through a 30% sucrose cushion, then on a 20–60% continuous sucrose gradient, each for 2.5 h at 100,000× g. We fractionated the gradient into 37 310-µl fractions and determined the virus titer (hemagglutination units (HAU) per ml) in each fraction by standard methods [19]. We combined virus-containing gradient fractions 13–30 into four separate sequential pools and subjected them to the final pelleting step at 100,000× g for 2.5 hrs. We resuspended the pool-4 pellet, corresponding to gradient fractions 21–30 enriched in long virions (Figure 4C), at ∼6 mg/ml of viral protein in HNE20 and used this preparation in most of the experiments described in this study. We used the pool-2 pellet, corresponding to gradient fractions 16–18 enriched in short virions, to compare relative incorporation of M2 into short and long virions (Figure 4C).

#### Labeling

We incubated 3 µl of purified Udorn-X31HA with 12 µl of 80 mM fluorescein solution in HNE5 (5 mM HepesNaOH pH 7.4, 145 mM NaCl, 0.2 mM EDTA) for 2 days at 4°C protected from light. We removed excess dye on a 5-ml PD-10 desalting column (GE-Healthcare) against HNE50 (50 mM HepesNaOH pH 7.4, 137 mM NaCl and 0.2 mM EDTA) and used the most concentrated 250-µl virus-containing fraction. To load virions with SRB we followed the previously described protocol [15], except that again we used HNE50.

### Single-virion fusion/internal acidification

Glass coverslips were cleaned extensively, then silanized and functionalized with a thin film of dextran polymer as previously described [15], except that the final cleaning step preceding surface functionalization consisted of 3 min-long plasma etching at 500 mTorr O_2_ and 75 W (March Plasmod Plasma Etcher, March Instruments, Inc.).

We constructed PDMS flow cells containing 0.5 mm wide and 70 µm high channels (5 per flow cell) as follows. Silicon 3″ wafer P(100) test grade (UniversityWafer) was extensively cleaned by sonication in acetone (5–10 minutes), then methanol (1–2 minutes), then rinsed in isopropanol, dried under stream of air and baked at 200°C for 5 minutes. The final cleaning step consisted of 4 min-long plasma etching at 120–140 O_2_ and 200 W. Negative photoresist SU-8 2025 (MicroChem) was spin coated onto a cleaned silicone wafer at 1800 rpm for 45 sec (speed was ramped up at 100 rpm/sec for 5 sec, then 300 rpm/sec until the target speed), then prebaked on a hotplate at 65°C for 3 minutes, then the temperature of the hotplate was gradually increased by 3 min-long 5°C increments to 95°C for the final soft-bake step of 6 minutes. The photoresist was exposed through a mask defining the channels for 19 sec at 365 nm and 160 mJ/cm^2^, then subjected to post-exposure bake on a hotplate set at 65°C for 2 minutes, then 95°C for 6 minutes, then cooled prior to developing using SU-8 developer (MichroChem) according to manufacturer's recommendations. Sylgard 184 silicon elastomer (PDMS) (Dow Corning) was prepared by mixing and degassing 50 g base and 5 g curing agent in ARE-250 (Thinky) at 1500 rpm for 2 minutes then 800 rpm for 2 minutes. PDMS was then poured on top of the prepared mold, degassed under vacuum for 30 minutes and baked at 65°C for 1.5 hours.

Fluorinated ethylene propylene tubing (Teflon FEP) (8 cm long, I.D. = 0.2 mm) (Upchurch Scientific) was inserted into one end of each channel and into a tube with solution to be introduced into the channel. Polyethylene tubing (30 cm long, I.D. = 0.76 mm) (Intramedic) was inserted into the opposite end of each channel and connected to a syringe pump (Harvard Pump 11; Harvard Apparatus).

Liposomes consisted of 4∶4∶2∶0.1∶2×10^−4^ ratio of 1,2,dioleoyl-sn-glycero-3-phosphocho- line (DOPC) (Avanti Polar Lipids), 1-oleoyl-2-palmitoyl-sn-glycero-3-phosphocholine (POPC) (Avanti Polar Lipids), cholesterol (Avanti Polar Lipids), bovine brain disialoganglioside GD1a (Sigma), and N-((6-(biotinoyl)amino)hexanoyl)-1,2- dihexadecanoyl-sn-glycero-3-phosphoethanolamine (biotin-X DHPE) (Molecular Probes). They were prepared as previously described [15], except they were resuspended in HNE50 at 20 mg/ml and extruded through a polycarbonate membrane filter with pore sizes of 200 nm.

We mounted a flow cell on the microscope stage and loaded liposomes at the rate of 0.04 ml/min for 1 min, after which the flow was stopped and the liposomes incubated for a minimum of 15 min. The unattached liposomes were washed by a 2-min flow with HNE50. To load virus particles the virus containing solution was pumped at the rate of 0.04 ml/min for 30 sec, the flow was stopped and attachment allowed to proceed for 5–15 min. 30 µg/ml fluorescein-labeled streptavidin was then introduced and incubated for 10 min before washing with HNE50. To initiate fusion/internal virion acidification citrate buffer (10 mM citric acid, 140 mM NaCl and 0.1 mM EDTA) at the appropriate pH was introduced at the rate of 0.07 ml/min. For rimantadine treatment, virus was preincubated with 10 µM rimantadine for 30 min, and all subsequent buffers contained rimantadine at the same concentration. All experiments were performed at 23°C.

### Imaging

We used the microscope configuration described previously [15] with important modifications. The power of the 488 nm laser used to excite fluorescein was adjusted either to 0.15 µW (low light) or to 3 µW (high light) and that of the 568 nm laser used to excite SRB either to 0.2 µW (low light) or to 4 µW (high light) over the same ∼70×140 µm^2^ area. Acquisition times were either 0.5 sec or 1 sec (low light) or 100 msec (high light).

### Derivation of onset and dissipation times

The following model admits a finite transition width (see Figure 1C):
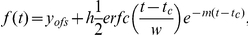
where *h* is the height of the intensity step, *w*, the half-width (in sec) of the transition, *t_c_*, the time at which the transition is half complete, *y_ofs_*, the residual intensity after dissipation, and *m*, a decay rate (typically slower than 1/*w*); *erfc(⋅)*, the complementary error function, is the integral of a Gaussian, defined as
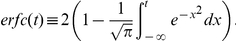
It captures the shape of the dissipation step and its finite transition time, as defined by the width, 2*w*, of the underlying Gaussian. The exponential factor, *exp[−m(t−t_c_)]*, is included to account for signal dissipation that sometimes begins before the sharp drop in fluorescence and that we interpret as due to photobleaching (see Figure 2A for an example of one such particle trace). This factor does not significantly affect *t_c_* and *w*, the parameters defining onset and dissipation times. Even when the presumed photobleaching is rapid, it still seems reasonable to use the exponential factor to separate the nonspecific contribution to signal decay from the contribution captured by the Gaussian transition component. We derive onset time for each virion as the interval from the pH drop time, *t_o_* (estimated as described [15]), to onset of dissipation, defined as *(t_c_−w)*, i.e. Δ*t_on_* = *t_o_*−*(t_c_−w)*. Similarly, we define the dissipation time ( = 2*w*), as the interval from onset at *t* = *(t_c_−w)* until *t = (t_c_+w)*. Note: *w* is equivalent to the standard deviation of the underlying Gaussian distribution, times 2^1/2^.

### Fitting of onset times and estimate of the light-induced rimantadine-insensitive subpopulation in the presence of rimantadine

The gamma distribution,
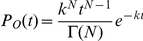
describes the statistics of a process of *N* steps, each with rate constant *k*, required (either in series or in parallel) for a measured event (in this case onset of fluorescence dissipation). We used this expression to fit all onset-time distributions in Figure 3A except fluorescein-signal dissipation in the presence of rimantadine, for which we used a sum of two gamma distributions:
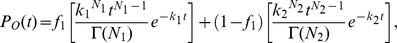
where f_1_ is the fraction of the overall distribution contributed by subdistribution 1. We first obtained values for k and N from a gamma fit of the light-induced rimantadine-insensitive fluorescein subpopulation in the absence of rimantadine (Figure 3A, plot iii and table I), then fixed those values as k1 and N1 in the dual gamma fit of the overall distribution of fluorescein-signal dissipation in the presence of rimantadine to derive parameters k2, N2 and f1 (Figure 3A, plot iv and table II). With these parameters, we can then subtract the light-induced, rimantidine-insensitive component (Figs. 3A and 3B, plot iii) from the overall distributions of both onset (Fig. 3A, plot iv) and dissipation times (Fig. 3B, plot iv) of fluorescein signal in the presence of rimantidine. We adjusted the areas of the distributions to be subtracted by f1 (0.27) or by f1 ± fit-derived error (0.33, 0.21) and subtracted them from the corresponding overall distributions with area normalized to 1 (Figs. 3A and B, plots vi, using 0.27).

### Negative stain electron microscopy

We applied 3 µl of the appropriate virus fraction to electron microscope grids (400 square mesh copper Gilder grid (Ted Pella), coated with Collodion (EMS) and carbon, and glow discharged using EMS100 (EMS) before applying sample) for 30 seconds. The grid was rinsed once with a drop of PTA stain (2% phosphotungstic acid, pH 7.5–8, filtered through 0.2-µm supor membrane filter (Pall Life Sciences)) then incubated with a fresh drop of PTA stain for 30 seconds and dried under mild suction from a vacuum port. Samples were imaged on Philips CM10 transmission electron microscope.

### SDS-PAGE and immunoblotting

For detection of virus particle-associated bands, we used step-gradient 10–16% Tris/tricine gels with 4% stacker. For anti-HA1 blots, proteins from the 10% gel portion were transferred to 0.45 µm nitrocellulose membrane (Immobilon, Millipore) for 1 h at 100 V in 1X transfer buffer (20 mM Tris base, 20 mM glycine). For anti-M2 blots, proteins from the 16% gel portion were transferred to 0.2 µm polyvinylidene fluoride membrane (Immobilon, Millipore) for 15 h at 10 mA in 1X transfer buffer plus 20% methanol. After transfer of proteins for M2 detection, membranes were fixed with 0.2% glutaraldehyde in PBS for 30 min and then washed extensively with PBS. For detection of HA1, antibody incubations were performed in PBS, 5% milk and 0.05% Tween-20. No Tween-20 was used in blots for M2 detection. Anti-HA1 antibody (6e2) (Cell Cignaling) was used at 1∶5000 dilution. Anti-M2 antibody (14C2) (Thermo Scientific) was used at 1∶1000 dilution. Mouse-specific horseradish peroxidase-conjugated IgG (Promega) was used in secondary detection at 1∶10000 (for HA1 detection) or 1∶5000 (for M2 detection). Antibody binding was detected with ECL Plus Western Blotting Detection Reagents (GE Healthcare) and the membrane was exposed to fluorography film (GeneMate, ISC Bioexpress).

### Particle-length measurement

We wrote a user-interfaced MATLAB (r2010a) routine to estimate virion lengths from electron micrographs. Sequential electron micrographs are loaded, and the user is prompted to click on each end of the scale bar and to enter the length it represents. The user then clicks on the ends of each virion, and for elongated virions on additional points along the virion axis. The long-axis length for each virion is then scaled appropriately using entered scale bar information. The combined virion-length information from multiple electron micrographs is displayed as a histogram (Figure 4B, top right).

### Estimate of the number of titratable groups in the virion interior

#### Internal viral protein-associated titratable groups

We used previously published estimates for the number of NP proteins per bp of viral genomic RNA (∼24) [20], the total length of A/Udorn/72 genome (13,430 bp), and the ratio of M1 to NP in virions (∼2.7) [21] to estimate the number of NP and M1 molecules per virion. We multiplied the derived number of virion-associated M1 by 2.7 to account for the elongated virion morphology of the virus preparation used in our kinetic measurements (mean length is 270 nm; Figure 4B, top right). We assumed that all histidine residues (5 in each M1 and NP) bind protons with a pK_a_ spanned by the starting and target pH of our experiments. We derive the value of ∼23,000 M1- and NP-associated titratable groups.

#### Internal fluorescein molecules

We determined the hemagglutination (HA) titer of the fluorescein-loaded virus preparation by standard methods [19], then estimated the number of virus particles per volume of labeled virus stock (nvp) from log nvp/HA = 7 [22]. We lysed a labeled-virus aliquot with 0.5% triton X-100 to release internal fluorescein and estimated the number of fluorescein molecules in this solution by comparing relative fluorescence with a series of standards of known fluorescein concentration using a fluorescence plate reader (Safire2, Tecan). To determine the upper limit for the number of internal fluorescein molecules, we assumed that there is no free fluorescein in our labeled-virus preparation. We derive the value of ∼8,000 fluorescein molecules per virion, yielding a total of ∼31,000 internal virion-associated titratable groups.

## Supporting Information

Figure S1 **Kinetics of internal virion acidification of a short virus particle preparation.** A) Proton-concentration dependence of onset (left) and dissipation rates (right). Error bars are propagated from the standard error of the mean (SEM) of measured onset and dissipation times as SEM(mean time)/(mean time)^2^. B) *Left* – a representative negative-stain electron micrograph of the egg-grown virus preparation. *Right* – the egg-grown virion length histogram distribution as estimated from multiple negative stain electron micrographs (see Methods).
(TIF)
