# Supplementary figures and images for: Kinetics of Proton Transport into Influenza Virions by the Viral M2 Channel
Source: PLoS One. 2012 Mar 6;7(3):e31566. doi: 10.1371/journal.pone.0031566 (PMC3295812; doi:10.1371/journal.pone.0031566)

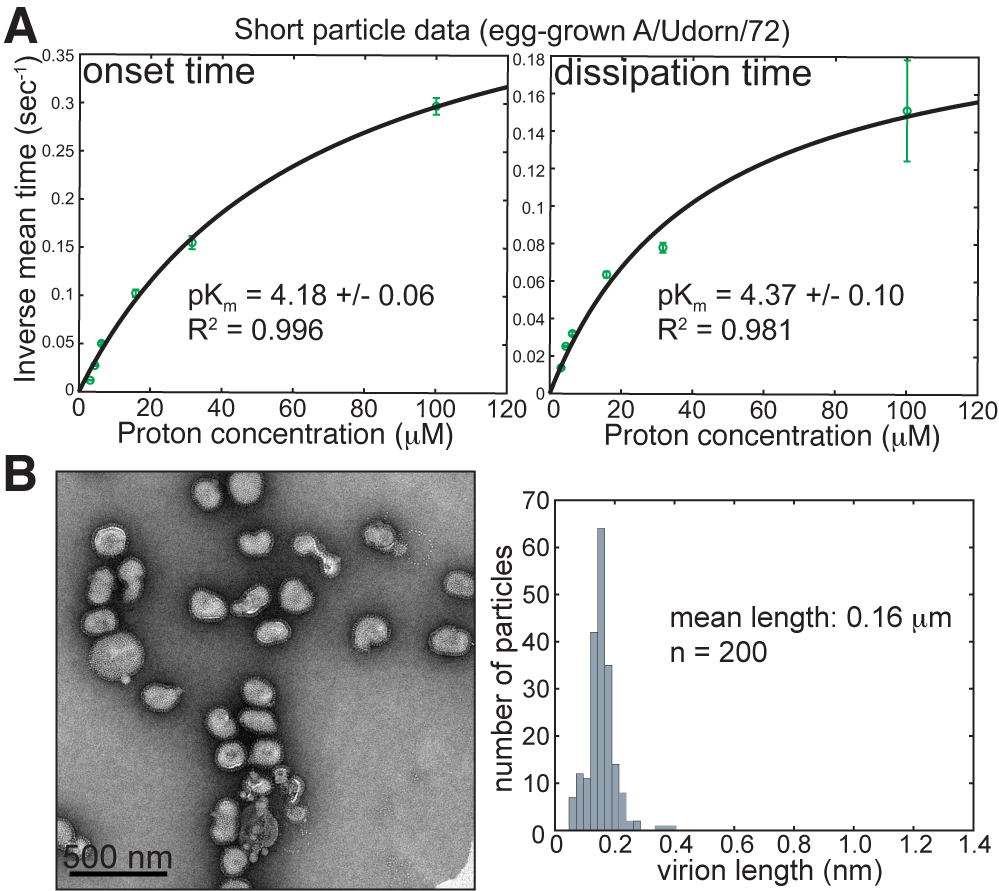

Supplement: Figure S1 — Kinetics of internal virion acidification of a short virus particle preparation. A) Proton-concentration dependence of onset (left) and dissipation rates (right). Error bars are propagated from the standard error of the mean (SEM) of measured onset and dissipation times as SEM(mean time)/(mean time)2. B) Left – a representative negative-stain electron micrograph of the egg-grown virus preparation. Right – the egg-grown virion length histogram distribution as estimated from multiple negative stain electron micrographs (see Methods). (TIF) [file pone.0031566.s001.tif]
